# Development of Islet Antigen 2 (IA2) Antibodies Post-COVID-19 Infection: A Sign of Autoimmunity or Latent Autoimmune Diabetes Mellitus in Adults (LADA)?

**DOI:** 10.7759/cureus.40971

**Published:** 2023-06-26

**Authors:** Nick Lee, Praveen Prabhu, Samyukta Swaminath, Sahar S Amini

**Affiliations:** 1 Internal Medicine, Nova Southeastern University Dr. Kiran C. Patel College of Osteopathic Medicine, Davie, USA

**Keywords:** gad 65, diabetes mellitus type 1.5, diabetes mellitus type 2, latent autoimmune diabetes in adults (lada), covid-19

## Abstract

Further antibody tests and laboratory studies showed that the patient met the criteria for latent autoimmune diabetes in adults (LADA). In this case report, we will review the diagnostic workup and management of LADA in an individual following his COVID-19 infection.

## Introduction

Latent autoimmune diabetes in adults (LADA) is a progressive form of autoimmune diabetes mellitus characterized by the presence of islet beta cell autoantibodies, lack of dependence on insulin, and older age at diagnosis [1]. LADA, the most common form of adult-onset autoimmune diabetes mellitus, has features of both type 1 and type 2 diabetes mellitus. According to the Immunology for Diabetes Society, the three criteria for the diagnosis of LADA, also known as diabetes type 1.5, include age greater than 35 years, positive autoantibodies to islet beta cells, and insulin independence for at least six months after the initial diagnosis. The insulin requirement, or the lack thereof, is debated since the choice of insulin as treatment is highly physician-dependent. The reason for this ambiguity stems from the pathophysiology of LADA being similar to T1DM, but since the immune destruction occurs at a slower rate, the presentation more closely mimics T2DM and was treated with non-insulin medications [2]. Most patients diagnosed with LADA are positive for one of the following autoantibodies: islet cell antibody (ICA), glutamic acid decarboxylase antibody 65 (GAD65), or islet antigen 2 (IA2) [3].

Genetic factors may be seen as the primary determinant for the cause of LADA. Carriers of specific human leukocyte antigen (HLA) haplotypes have a higher risk of acquiring LADA in type 1 diabetes mellitus (T1DM) because these HLA genes code for major histocompatibility antigens (MHC), which have an immunoregulatory function. However, only limited studies show that LADA may have similar lifestyle risk factors with type 2 diabetes mellitus (T2DM), such as excess body weight, heavy smoking, intake of two or more sweetened beverages daily, and others that influence insulin sensitivity [4]. This case is pertinent because this patient had no genetic predisposition to T1DM and was diagnosed with the disease post-exposure to coronavirus disease 2019 (COVID-19), despite well-controlled sugar levels and a balanced diet.

## Case presentation

A 46-year-old male tested positive for COVID-19 in December 2021 during a routine nasopharyngeal nucleic acid amplification test at work. The patient was up to date with his severe acute respiratory syndrome coronavirus 2 (SARS-CoV-2) vaccinations and booster, all of which were manufactured by Pfizer. His past medical history included type 2 diabetes diagnosed at age 35, major depressive disorder, Klinefelter syndrome, and low testosterone. He had no pertinent family history and no autoimmune history. His medications included semaglutide 0.5 mg subcutaneously once a week and vortioxetine 10 mg daily. His prior laboratory data 7 months ago showed his hemoglobin A1c (HgA1c) at 11.9 with the current medication regimen. Two days following his positive COVID-19 test, he experienced intractable vomiting and went to the emergency department (ED). At the ED, his vitals were as follows: body mass index (BMI) 37, Temp 104.2°F, and heart rate (HR) 170 beats per minute. Laboratory data were significant for HgA1c 14.0 and blood sugar level 363 mg/dL. He was given 11 units of regular insulin via IV push to bring down his glucose levels down to 214 mg/dL as well as 600 mg of casirivimab-imdevimab each equaling 10 mL infused over 30 min. After spending one day in the ED, he was discharged and advised to follow up outpatient with his primary care provider and continue symptomatic management. 

After one day following discharge from the ED, he experienced consistent hyperglycemia with blood sugar levels ranging between 300 and 400 despite dietary changes such as eliminating carbohydrates for one week and continuing his routine semaglutide 0.5 mg dose. Ten days after his ED visit, he was placed on insulin degludec and reached a blood sugar of 80-120 with up-titrating degludec to 50 units/day. His BMI after implementing these changes was 31, which was a couple of months after reaching the optimal titration of insulin degludec 50 units/day.

Subsequently, the patient had the following laboratory values (Table 1): GAD65 was <5 (within normal limits), C-peptide level 2.38 (within normal limits), negative HLA-DR, and elevated IA2 autoantibodies at 20.6 (normal range <5.4). With a positive result of IA2 autoantibodies, the patient met the criteria for LADA. His normal C-peptide level and negative HLA-DR indicated endogenous insulin production with no predisposition for autoimmunity.

**Table 1:** Laboratory values. IA2,  islet antigen 2

Parameters	Lab value	Reference range
GAD65	<5 nmol/L	<0.02 nmol/L
C-peptide	2.38 ng/mL	0.5-2.0 ng/mL
IA2 autoantibodies	20.6 DK units/mL	<5.4 DK units/mL

## Discussion

The first part of this analysis requires understanding that the role the coronavirus may have had in the production of an autoimmune response. In a study that analyzed patients hospitalized with COVID-19, researchers screened for autoantibodies in blood samples from almost 150 people hospitalized with COVID-19 and 41 healthy volunteers. Though the sample size is small, approximately 50 people with COVID-19 had blood samples drawn on more than one day, including the day they were hospitalized. These samples showed that 20% of these patients did not have autoantibodies when they were first admitted but developed them through the course of the disease [5]. The mechanism behind this phenomenon was unclear, but it shows that the presence of autoantibodies can develop post-COVID-19 infection, as shown in our patient. One possible mechanism behind the coronavirus causing an autoimmune reaction could be its relationship with the angiotensin-converting enzyme 2 (ACE2) as its primary receptor to infect cells [6-7].

The ACE2 receptors are expressed in both the exocrine pancreatic glands and islet cells. Once the spike protein of the virus binds to ACE2, a transmembrane protease serine 2 (TMPRSS2) primes the spike protein and facilitates its entry. Conversely, when ACE2 receptors are blocked through the binding of the virus, angiotensin II levels increase. The increased levels of angiotensin II sustain the activation of Na+/H+ exchanger 2(NHE2), promoting reactive oxygen species development. This sustained NHE2 increases oxidative stress and ultimately causes insulin resistance and beta cell injury [6-7]. 

There are certain limitations with the patient presentation and analysis of this case report. Excessive amounts of inflammation boost the production of autoantibodies that existed prior to the infection at low levels. Our patient possibly had the IA2 antibodies before becoming infected, but confirmation of that is unavailable since the testing was not performed. 

Latent autoimmune diabetes in adults and classical T1DM may present similarly; however, whereas T1DM often presents rapidly, LADA progresses slower as beta cell function is gradually lost. Hence, patients may respond initially to non-insulin glucose-lowering agents, but that response will eventually decrease [8]. As a result, there should be more efforts in elucidating the diagnosis between LADA, T1DM, and T2DM before starting and continuing a patient on non-insulin therapeutics. Without the appropriate lifestyle modifications, non-insulin therapeutics will have decreased effectiveness, and the patient will eventually require insulin.

Further workup in our patient includes monitoring auto-antibodies levels to see if there are any other changes from the time of diagnosis. In addition, screening for auto-antibodies in those with either genetic predispositions to type 1 diabetes or current endocrinologic disease is warranted. For example, a systematic review by Gracia-Ramos, Martin-Nares, and Hernández-Molina reported vasculitis and arthritis as the primary rheumatologic diseases caused by COVID-19. Studies showed that 35.6% of patients with COVID-19 tested positive for antinuclear antibodies (ANA), 25% for anti-Ro/SSA antibodies, 19% for rheumatoid factor, and 11% for lupus anticoagulant antibodies [9]. There has not been formal research to quantify the development of IA2, GAD65, and ICA antibodies in patients with COVID-19. This is an area of future research that may help provide better insight into autoimmunity post-COVID-19 infection. 

## Conclusions

This case suggests that SARS-CoV-2 has the potential to induce an autoimmune reaction against pancreatic beta cells in patients who have type 2 diabetes without having the genetic predisposition for type 1 diabetes. However, the diagnosis of LADA in our patient is inconclusive because antibody testing was not performed prior to his COVID-19 infection. Case-based empiric testing of those with both a family history of auto-immune diseases and exposure risks for COVID-19 is suggested to help elucidate whether large-scale testing is warranted. Large-scale studies could potentially help clarify the causality and mechanism behind why such a reaction occurs in patients. Specifically, testing the number of autoimmune antibodies in a randomized clinical trial involving patients with T2DM would answer some of the pending questions.
